# Acute Pancreatitis in Individuals with COVID-19: A Case Report and Critical Review of Literature

**DOI:** 10.1155/2022/1275287

**Published:** 2022-06-18

**Authors:** Arsia Jamali, Khashayar Nemovi, Betul N. Bayraktutar, Adam Farzaneh, Kevin Chan, Carlos Ramirez, Elika Shahkamrani, Anil Perumbeti

**Affiliations:** ^1^Eisenhower Medical Center, Rancho Mirage, CA, USA; ^2^Tufts Medical Center, Tufts University School of Medicine, Boston, MA, USA

## Abstract

Involvement of gastrointestinal tract has been reported in individuals diagnosed with COVID-19. Herein, we report a case of 65-year-old woman with type 2 diabetes mellitus, hypertension, and end-stage renal disease undergoing hemodialysis who was initially diagnosed with COVID-19 on a screening test. During the course of the disease, her respiratory symptoms remained mild; however, she developed acute pancreatitis leading to severe hypertension and hyperosmolar hyperglycemic state. During the hospitalization and treatment of acute pancreatitis, hyperglycemia, and hypertension, her condition improved and she was discharged in stable condition.

## 1. Introduction

Gastrointestinal symptoms including diarrhea, nausea, vomiting, abdominal pain, and belching/reflux have been widely reported in patients diagnosed with COVID-19 [1, 2]. Notably, some recent reports have suggested that COVID-19 might be associated with abdominal pain, pancreatic injury, or acute pancreatitis [3–5]. However, in-depth assessment of the reports indicates that many of the reported cases of acute pancreatitis in the literature lack accurate diagnosis of acute pancreatitis based on modified Atlanta criteria [6]. Herein, we report a case of acute pancreatitis, leading to severe hypertension and hyperosmolar hyperglycemic state in a patient with SARS-CoV-2 infection.

## 2. Case Presentation

A 65-year-old Hispanic woman with history of type 2 diabetes mellitus, hypertension, end-stage renal disease, hypothyroidism, and morbid obesity was diagnosed with COVID-19 based on a routine PCR test at her hemodialysis facility. About two weeks later, she presented to our hospital with confusion, intractable nausea, vomiting, increased cough, and shortness of breath for 2 days. While the patient has been on atorvastatin, carvedilol, bumetanide, hydralazine, clonidine, detemir insulin, glipizide, glyburide, and levothyroxine, she has not been able to take her medications in the last 2 days due to severity of nausea and vomiting and missed her dialysis sessions. Her social history was negative for smoking or intake of alcoholic beverages. On arrival, her vital signs indicated blood pressure of 222/94 mmHg, pulse rate of 67/min, respiratory rate of 15/min, and temperature of 36.6°C with SpO2 of 96% on 2 L/min nasal cannula. Initial lab work indicated blood glucose of 1661 mg/dL with bicarbonate of 30.6 mmol/L, normal anion gap, and BNP of 1,810.0 pg/mL (Table 1). The patient was started on insulin and nicardipine drips which improved her blood pressure and blood glucose level. Chest X-ray showed bilateral infiltrates with pulmonary congestion, and the patient was treated with azithromycin and ceftriaxone for COVID-19 pneumonia. Due to hyperglycemia, steroids were not started at the presentation. For treatment of severe hypertension and pulmonary edema, hemodialysis was performed. After improvement in her mental state, she reported severe epigastric abdominal with radiation to back, escalating since one day prior to hospitalization. Serum lipase was elevated (1,142 U/L; reference range: 11–82) and CT scan demonstrated diffuse edematous enlargement of the pancreas with moderate pancreatic infiltrative stranding and fluid. No gallstones, biliary dilatation, or biliary filling defects were observed. Further etiologic assessment of pancreatitis was negative for alcohol intake, hypercalcemia, severe hypertriglyceridemia, or offending medications. With continuing supportive management, patient tolerated oral intake. Five days later, she was discharged upon decrease in lipase to 261 U/L, improved blood pressure, improved glycemic control, and return to normal oxygenation.

## 3. Discussion

Considering the presenting symptoms, signs, and the lab work, it seems that the patient has been experiencing acute pancreatitis, which due to intractable nausea and vomiting led to medication non-adherence and subsequently severe hypertension and hyperosmolar hyperglycemic state.

In individuals with COVID-19, elevated pancreatic enzymes were reported in about 12.1–17% of cases in different reports [3–5, 7]. Further, increased serum amylase and lipase to more than three times of upper normal limit has been reported in cases without diagnosis of acute pancreatitis [3, 5].

Although obstructing common bile duct stones, alcohol consumption, hypertriglyceridemia, medications, traumas, and severe medical conditions comprise the majority of the underlying causes of acute pancreatitis, infectious etiologies may account for about 10% of encounters [8]. In such cases, paramyxoviruses such as mumps and measles viruses, enteroviruses, namely, Coxsackie B viruses and hepatitis A virus, and Epstein–Barr virus from Herpesviridae are the most well-known etiologies of acute pancreatitis [8, 9]. Currently, about fifty cases of acute pancreatitis have been reported in the literature in individuals with COVID-19. Nevertheless, there are several shortcomings in many of these reports which makes it difficult to assume causal relationship between SARS-CoV-2 and acute pancreatitis. In brief, in some of the reports, cases do not meet the diagnostic criteria of acute pancreatitis based on Atlanta classification [10] (Table S1). In many reports, common causes of acute pancreatitis have not been clearly ruled out (Table S1). Further, in some of the cases, diagnosis of acute pancreatitis and hospitalization preceded diagnosis of COVID-19 or positive SARS-CoV-2 test, which may suggest that COVID-19 has occurred as a nosocomial infection rather than serving as the etiology of the acute pancreatitis, as reported in 5 individuals with acute pancreatitis [11]. However, in our case, (1) COVID-19 was diagnosed about two weeks prior to hospitalization and (2) the major etiologies of acute pancreatitis including common bile duct stone, alcohol consumption, hypercalcemia, hypertriglyceridemia, trauma, and offensive mediations were clearly ruled out.

Similar to other viral etiologies underlying acute pancreatitis, it is not clear if involvement of the pancreas is directly related to invasion of the virus to the pancreatic tissue and subsequent direct cytopathic effect of local SARS-CoV-2 replication or it is a consequence of immune reaction to the virus, leading to destruction of the pancreatic tissue as a naïve bystander. Of note, SARS-CoV-2 enters the host cells through angiotensin-converting enzyme-2 (ACE-2) receptor and transmembrane serine protease 2 (TMPRSS2). Single-cell RNA analysis as well as protein expression on autopsies has shown that pancreatic ductal and acinar cells co-express these molecules, suggesting that SARS-CoV-2 can enter pancreatic ductal cells [12–14]. Notably, messenger RNA level of ACE2 might be higher in the pancreas than in the lungs [7]. Also, expression of ACE2 is increased in diabetic murine models and diabetic donors [15, 16]. Coronaviruses were reported to cause acute pancreatitis in ferrets and pigeons [17, 18]. In cases of SARS-CoV-2 infection, autopsies revealed pancreatitis without clinical suspicion of pancreatitis in 25–45.5% of cases [19, 20]. Similarly, SARS-CoV-2 PCR was found positive in pancreas pseudocyst [21] suggesting that the virus may be directly present in the pancreatic tissue during the course of acute pancreatitis. Nevertheless, further studies including experimental models are warranted to evaluate how pancreas might be damaged during COVID-19 infection and associated inflammatory response.

## Figures and Tables

**Table 1:** Laboratory findings.

	Case	Reference range
Sodium (mmol/L)	126	136–145
Potassium (mmol/L)	5.0	3.5–5.1
Chloride (mmol/L)	87	98–107
Bicarbonate (mmol/L)	30.6	23.0–29.0
Anion gap (mmol/L)	8	3–11
Glucose (mg/dL)	1,661	70–105
BUN (mg/dL)	69	7–25
Creatinine (mg/dL)	3.5	0.6–1.2
Albumin (g/dL)	2.9	3.5–5.7
Calcium (mg/dL)	8.4	8.6–10.3
Total bilirubin (mg/dL)	0.3	0.3–1.0
Alkaline phosphatase (IU/L)	131	34–104
AST (U/l)	17	13–39
ALT (IU/L)	9	7–52
BNP (pg/mL)	1,810.0	1.0–100.0
Beta-hydroxybutyrate (mmol/L)	<0.10	0.02–0.27
Lactate (mmol/L)	1.9	0.5–2.2
CRP (mg/dL)	7.7	<0.1
Ethanol (mg/dL)	<10	
Triglycerides (mg/dL)	385	<150
Urine rapid drug screen	Negative	
Ferritin (ng/mL)	3,230.0	11.0–306.8
D-Dimer, mg/L fibrinogen-equivalent units	1.19	0.0–0.48
WBC, x10^9^ cells/L	7.9	3.8–10.8
Hemoglobin (g/dL)	9.3	12.0–16.0
MCV, fL	105.5	80.0–99.0
Platelet count, x10^9^ platelets/L	260	150–450

AST, aspartate transaminase; ALT, alanine transaminase; BNP, B-type natriuretic peptide; BUN, blood urea nitrogen; CRP, C-reactive protein; MCV, mean corpuscular volume; WBC, white blood cell.

## Supplementary Materials

Supplementary Materials Table S1: reported cases of acute pancreatitis in individuals with COVID-19.
