# Supplementary material for: Acute Pancreatitis in Individuals with COVID-19: A Case Report and Critical Review of Literature
Source: Case Rep Med. 2022 Jun 18;2022:1275287. doi: 10.1155/2022/1275287 (PMC9233612; doi:10.1155/2022/1275287)
Supplement: Supplementary Materials — Table S1: reported cases of acute pancreatitis in individuals with COVID-19. [file 1275287.f1.docx]

Table S1. **Reported cases of acute pancreatitis in individuals with COVID-19.**

|  | Age | Gender | Abdominal Pain | Pancreatic Enzymes | Imaging | Onset of Pancreatitis | Etiologies of Pancreatitis Ruled out | Comments | Reference and  PMID |
| --- | --- | --- | --- | --- | --- | --- | --- | --- | --- |
| Case: 1 | 47 | F | Not reported | Amylase>  1,500 U/L | Supportive U/S findings | 1 week after respiratory symptoms | Cholelithiasis  Hypercalcemia  Hypertriglyceridemia  Alcohol consumption |  | [1]  [32387082](https://www.ncbi.nlm.nih.gov/pubmed/32387082) |
| Case: 2 | 68 | F | Epigastric | Amylase: 934 U/L | None | At the time of systemic symptoms | Hypercalcemia  Hypertriglyceridemia | Other etiologies not sufficiently ruled out |  |
| Case: 3 | 71 | M | Not reported | Amylase: within normal limits | None | At the time of systemic and respiratory symptoms | Alcohol consumption | Questionable diagnosis of acute pancreatitis |  |
| Case: 1 | 59 | F | Abdominal | Not reported | Supportive CT scan findings | 10 days after initial diagnosis of COVID-19 | Cholelithiasis  Alcohol consumption | Questionable diagnosis of acute pancreatitis; other etiologies not sufficiently ruled out | [2]  [32339257](https://www.ncbi.nlm.nih.gov/pubmed/32339257) |
| Case: 1 | 36 | F | Epigastric; stabbing; radiating to back | Amylase and lipase>3× normal limit | Supportive CT scan findings | 6 days after upper respiratory symptoms | Hypertriglyceridemia | Other etiologies not sufficiently ruled out | [3]  [32444169](https://www.ncbi.nlm.nih.gov/pubmed/32444169) |
| Case: 1 | 36 | F | Epigastric; belt-like | Amylase: 718 U/L; lipase: 631 U/L | None | 11 days after systemic and respiratory symptoms | Cholelithiasis  Hypercalcemia  Hypertriglyceridemia  Coxsackie virus  Herpes virus 1 Herpes virus 2  Cytomegalovirus  Hepatitis B virus  Hepatitis C virus  Human immunodeficiency virus  Negative anti-nuclear antibody |  | [4]  32523925 |
| Case: 1 | 10 | F | Epigastric | Lipase: 365.7 U/L (upper limit: 70 U/L) | None | Not reported | Not reported | Other etiologies not sufficiently ruled out; diagnosis of COVID-19 was based on IgG | [5]  33309015 |
| Case: 2 | 16 | M | Epigastric | Lipase: 233.3 U/L (upper limit: 70 U/L) | None | After respiratory symptoms | Not reported | Other etiologies not sufficiently ruled out; intubated and extubated prior to pancreatitis |  |
| Case: 1 | 10 | F | Abdominal; diffuse; radiating to back and right lower quadrant | Lipase: 1,371 U/L | Supportive CT scan findings | No respiratory symptoms, 2 COVID-19 PCR tests were negative during hospitalization; serology became positive 2 weeks after discharge | Hypertriglyceridemia | Likely nosocomial COVID-19; other etiologies not sufficiently ruled out | [6]  32740530 |
| Case: 1 | 15 | M | Epigastric | Lipase: 233 U/L (upper limit: 39 U/L) | Supportive CT scan findings | A week after respiratory symptoms | Hypertriglyceridemia | Other etiologies not sufficiently ruled out | [7]  33075134 |
| Case: 2 | 11 | M | Periumbilical | Amylase 156 and 215 U/L; lipase: 582 and 953 U/L (upper limit 39 U/L) | CT scan: normal pancreas; findings suggestive of acute appendicitis | No respiratory symptoms; 6 days after general symptoms | Hypertriglyceridemia | Appendicitis was likely the etiology of increased pancreatic enzymes |  |
| Case: 3 | 16 | F | Epigastric; radiating to back | Lipase: 1,909 U/L (upper limit: 39 U/L) | Supportive U/S findings | 4 days after respiratory symptoms | Hypertriglyceridemia  U/S a single gallstone | History of pancreatitis |  |
| Case: 1 | 67 | M | Epigastric | Lipase: 5,295 U/L | Supportive imaging findings | 2 days prior to upper respiratory symptoms and a positive COVID-19 test | Cholelithiasis  Hypertriglyceridemia  Autoimmune etiology (by IgG4)  Offending medications  Trauma | Possibly alcohol related per history; likely nosocomial COVID-19 | [8]  33122538 |
| Case: 1 | 45 | F | Epigastric; sharp; radiating to back | Amylase: 364 U/L; lipase: 293 U/L | Supportive CT scan findings | About a week prior to respiratory symptoms | Alcohol consumption | Other etiologies not sufficiently ruled out; likely nosocomial COVID-19 | [9]  33234760 |
| Case: 1 | 57 | F | Epigastric; radiating to back | Lipase: 8,352 U/L | Supportive CT scan findings | 11 days after systemic symptoms | Cholelithiasis  Alcohol consumption  Trauma  Offending medications | Other etiologies not sufficiently ruled out | [10]  33363941 |
| Case: 1 | 40 | M | Epigastric; radiating to back | Lipase of 1,544 U/L (upper limit: 82 U/L) | Supportive CT scan findings | 1 day prior to respiratory symptoms | Cholelithiasis  Alcohol consumption | Hypertriglyceridemia was likely the etiology of acute pancreatitis (Triglyceride: 4,245 mg/dL) | [11]  [32604205](https://www.ncbi.nlm.nih.gov/pubmed/32604205) |
| Case: 1 | 67 | F | Epigastric | Amylase: 1,483 U/L | Supportive CT scan findings | No respiratory symptom reported | Cholelithiasis  Alcohol consumption  Hypertriglyceridemia  Hypercalcemia  Autoimmune etiology (by IgG4) |  | [12]  32900752 |
| Case: 1 | 47 | M | Abdominal diffuse; more severe in right lower quadrant | Amylase: 349 U/L; lipase>600 U/L | None | 5 days after respiratory symptoms |  | Questionable diagnosis of acute pancreatitis | [13]  [32691004](https://www.ncbi.nlm.nih.gov/pubmed/32691004) |
| Case: 1 | 70 | M | Abdominal | Amylase: 331 U/L; lipase:293 U/L | Supportive MRI findings | Not reported | Cholelithiasis  Alcohol consumption | Other etiologies not sufficiently ruled out | [14] 33321015 |
| Case: 2 | 47 | F | Not reported | Amylase: 175 U/L; lipase: 451 U/L |  | 3 days after respiratory/systemic symptoms | Cholelithiasis  Alcohol consumption | Other etiologies not sufficiently ruled out |  |
| Case: 1 | 7 | F | Abdominal | Lipase: 676 U/L (upper normal limit: 360 U/L) | Supportive U/S and CT scan findings | No respiratory symptoms | Not reported | Other etiologies not sufficiently ruled out. | [15]   - 32572339 |
| Case: 2 | - | F | Left lower quadrant, and epigastric | Lipase: 1,672 U/L | None | At the time of respiratory symptoms | Not reported | Other etiologies not sufficiently ruled out |  |
| Case: 1 | 56 | F | Epigastric | Amylase: 544 U/L; lipase: 2,993 U/L | Supportive CT scan findings | At the time of respiratory symptoms | Cholelithiasis  Alcohol consumption  Hypertriglyceridemia  Hypercalcemia  Offending medications  Trauma |  | [16] 32961108 |
| Case: 1 | 30 | M | Epigastric; radiating to the back | Lipase: 1,022 U/L  (upper normal limit<200 U/L) | Supportive CT scan findings | 2 days after respiratory symptoms | Alcohol consumption  Hypertriglyceridemia  Hypercalcemia |  | [17]   - 33391949 |
| Case: 1 | 32 | M | Epigastric; radiating to back | Amylase: 672 IU/L  Lipase: 721 IU/L | Supportive CT scan findings | 14 days after upper respiratory symptoms | Cholelithiasis  Alcohol consumption  Hypertriglyceridemia  Hypercalcemia |  | [18]  32662399 |
| Case: 1 | 61 | M | Abdominal; diffuse; sudden | Amylase: 142 U/L (upper normal limit: 100 U/L); lipase: 203 (upper normal limit: 67 U/L) | Supportive CT scan findings | 19 days after upper respiratory symptoms | Not reported | Other etiologies not sufficiently ruled out | [19]  32719021 |
| Case: 1-6 | 41-90 | 5 M, 1 F | Abdominal; diffuse in 3 cases | Amylase and lipase: 2 cases elevated>3× upper normal limit | No CT scan findings suggestive of pancreatitis | Not reported | Cholelithiasis  Alcohol consumption | Questionable diagnosis of acute pancreatitis; other etiologies not sufficiently ruled out | [20]  32497298 |
| Case: 1 | 72 | F | Not reported | Amylase 1,789 U/L; lipase: 1,247 U/L | Supportive CT scan findings | 2 days after ICU admission due to respiratory symptoms | Hypertriglyceridemia  Hypercalcemia | Other etiologies not sufficiently ruled out; might be related to taking Baricitinib | [21]  32797239 |
| Case: 1 | 38 | M | Epigastric | Lipase: 10,255 and 20,320 ukat/L | Supportive CT scan and MRI findings | 1 week after testing positive and 1 week after initial episode | Cholelithiasis  Hypertriglyceridemia  Hypercalcemia  Offending medications | Other etiologies not sufficiently ruled out | [22]   - 32833954 |
| Case: 1 | 72 | M | Abdominal | Lipase: 185 U/L (upper normal<67 U/L) | Supportive U/S and EUS findings | 8 days prior to respiratory symptoms | Cholelithiasis  Alcohol consumption | Questionable diagnosis of acute pancreatitis; other etiologies not sufficiently ruled out | [23]  32925503 |
| Case: 1 | 36 | F | Upper abdominal | Amylase: 710 U/l; lipase:  640 U/l | Supportive CT scan findings | 9 days after of respiratory symptoms | Cholelithiasis  Alcohol consumption,  Trauma | Other etiologies not sufficiently ruled out | [24]  33054297 |
| Case: 1 | 76 | F | Epigastric; radiating to hypochondriac regions | Amylase: 3,568 IU/L | Supportive U/S and CT scan findings | No respiratory symptoms reported | Cholelithiasis  Alcohol consumption | Other etiologies not sufficiently ruled out | [25]  32835111 |
| Case: 1 | 30 | F | Right upper quadrant | Amylase 193 U/L; lipase 291 U/L | Supportive CT scan findings | At the time or respiratory symptoms |  | Questionable diagnosis of COVID-19, since tests (IgM, IgG, and PCR) were negative; likely due to biliary pancreatitis based on U/S findings | [26]  [33137666](https://www.ncbi.nlm.nih.gov/pubmed/33137666) |
| Case: 1 | 78 | F | Epigastric; positional | Amylase: 185 iU/L;  lipase: 230 iU/L | Supportive U/S findings | 1 day prior to hospitalization and respiratory symptoms | Cholelithiasis  Alcohol consumption  Hypertriglyceridemia  Hypercalcemia |  | [27]  **33244387** |
| Case: 1 | 65 | F | Upper abdominal | Amylase: 192 U/L (upper normal limit: 140 U/L);  lipase: 283 U/L (upper normal limit: 60 U/L) | Normal CT scan findings | 7 days prior to respiratory symptoms which started on day 2 of hospitalization | Hypertriglyceridemia | Nosocomial COVID-19 might be considered; questionable diagnosis of acute pancreatitis; history of taking levofloxacin might have been the etiology of acute pancreatitis | [28]  32576441 |
| Case: 1 | 49 | F | Epigastric; radiating to back | Amylase: 501 (upper normal limit: 110); lipase (IU/L): 1,541 (upper normal limit: 160) | Supportive CT scan findings | 6 days after respiratory symptoms, on day 2 of admission | Cholelithiasis  Alcohol consumption  Hypertriglyceridemia  Hypercalcemia |  | [29]  32802606 |
| Case: 1 | 55 |  | No pain reported (patient was intubated and sedated) | Amylase: 252 U/L (upper normal limit: 132 U/L); lipase: 263 U/L (upper normal limit: 55 U/L) | Supportive CT scan findings | 14 days after hospitalization and 21 days after initial symptoms | Cholelithiasis  Hypertriglyceridemia  Hypercalcemia | Might be related to ICU admission | [30]  32963170 |
| Case: 1 | 68 | M | No pain reported | Amylase: 1,030 U/L; lipase: 2,035 U/L | Supportive CT scan findings | COVID-19 diagnosed on a routine PCR test- no respiratory symptoms | Cholelithiasis  Hypertriglyceridemia  Hypercalcemia | Nosocomial COVID-19 might be considered, since the patient was a nursing home resident | [31]  32968552 |
| Case: 1 | 53 | M | Abdominal | Lipase: 530 U/L | Supportive CT scan findings | At the time of respiratory symptoms- | Cholelithiasi  Alcohol consumption  Hypertriglyceridemia  Hypercalcemia | Likely due to autoimmune pancreatitis since IgG4 level was elevated to 361 mg/dL | [32]   - 32692733 |
| Case: 1 | 73 | M | Epigastric | Amylase: 59 U/L; lipase: 63 U/L | Supportive CT scan findings | 18 days after hospitalization due to respiratory symptoms |  | Cytomegalovirus pancreatitis likely; since cytomegalovirus duodenitis was diagnosed by endoscopic biopsy of the lesions and DNA test | [33]   - 32952971 |
| Case: 1 | 24 | M | Epigastric | Amylase: 391 U/L; lipase: 578 IU/L | Supportive CT scan findings | At the time of respiratory symptoms | Cholelithiasis  Alcohol consumption | Other etiologies not sufficiently ruled out | [34]  32685078 |
| Case: 1 | 26 | F | Epigastric | Lipase: 211 U/l (3.5× upper normal limit) | Supportive CT scan findings | No respiratory symptoms; although imaging evidence of lung involvement present | Cholelithiasis  Alcohol consumption  Hypertriglyceridemia  Hypercalcemia  Hepatitis B virus  Hepatitis C virus  Human immunodeficiency virus  Coxsackie viruses  Chlamydia  Mycoplasma  Negative antinuclear and anti-DNA antibodies |  | [35]  32492174 |
| Case: 1 | 20 | F | Epigastric; sharp | Amylase: 396 U/L (upper normal limit: 110 U/L); lipase: 916 U/L (upper normal limit: 160 U/L) | Supportive MRI findings | 5 days after respiratory symptoms | Cholelithiasi  Hypertriglyceridemia  Autoimmune etiology (by IgG4) | Other etiologies not sufficiently ruled out | [36]  33355431 |
| Case: 1 | 8 | M | Abdominal; diffuse | Moderate increase in pancreatic enzymes | Normal U/S | No respiratory symptoms reported at the time of abdominal symptoms | None reported | Questionable diagnosis of acute pancreatitis; other etiologies not sufficiently ruled out | [37]  33291619 |
| Case: 1 | 29 | M | Abdominal; diffuse; radiating to back | Amylase: 2,861 IU/L; lipase: 1,650 IU/L | Supportive U/S findings | No respiratory symptoms reported | Cholelithiasis  Hypertriglyceridemia  Hypercalcemia | Other etiologies not sufficiently ruled out | [38]  32934906 |
| Case: 1 | 47 | M | Epigastric; radiating to back | Amylase 6× upper normal limits; lipase: 22× upper normal limits | Supportive CT scan findings | 12 days after respiratory symptoms | Cholelithiasis  Alcohol consumption  Hypertriglyceridemia  Hypercalcemia  Offending medications  Trauma | Other etiologies not sufficiently ruled out | [39]  32531002 |
| Case: 1 | 58 | M | Epigastric | Amylase: 249 U/L (upper normal limit: 60); lipase>600 U/L | U/S did not visualize the pancreas | No respiratory symptoms | Alcohol consumption  Hypertriglyceridemia  Offending medications | Other etiologies not sufficiently ruled out | [40]  32923256 |
| Case: 1 | 36 | F | Epigastric | Amylase: 88 U/L; lipase: 875 U/L | U/S did not visualize pancreas | 5 days after respiratory symptoms | Cholelithiasis  Alcohol consumption  Hypertriglyceridemia  Trauma | Might have been related to taking dicloxacillin for mastitis | [41]  32537425 |
| Case: 1 | 58 | M | Abdominal; recurrent | Amylase: 792 (upper normal limit: 132 UI/L) | Supportive CT scan findings | 28 days after respiratory symptoms, 21 days after hospitalization | Cholelithiasis  Hypertriglyceridemia  Hypercalcemia | Might have been related to taking fravipiravir | [42]  33122537 |
| Case 1: | 29 | M | Reported as “typical” | Amylase: 77 U/L | Supportive CT scan findings | Not reported | Cholelithiasis  Alcohol consumption  Offending medications | Hypertriglyceridemia was likely the etiology of acute pancreatitis since triglyceride: 2,740 mg/dL | - [43] - 32497545 |
| Case: 2 | 41 | M | Reported as “typical” | Amylase: 149 U/L | Supportive CT scan findings | Not reported | Cholelithiasis  Offending medications | Questionable diagnosis of COVID-19 since test was negative;  alcohol consumption was likely the etiology of pancreatitis since ethanol consumption: 80 g/week. |  |
| Case: 3 | 42 | M | Reported as “typical” | Amylase: 378 U/L | Supportive CT scan findings | Not reported | Cholelithiasis  Offending medications | Questionable diagnosis of COVID-19 (test result was not reported); alcohol consumption was likely the etiology of acute pancreatitis since ethanol consumption was 400 g/week. |  |
| Case: 4 | 47 | M | Reported as “typical” | Amylase: 211 U/L | Supportive CT scan findings | Not reported | Cholelithiasis | Medications including sertraline or alcohol consumption might have been the etiology of pancreatitis (ethanol consumption: 50 g/week). |  |
| Case: 5 | 53 | M | Reported as “typical” | Amylase: 36 U/L | None | Not reported | Cholelithiasis  Alcohol consumption  Offending medications | Questionable diagnosis of COVID-19 since test was negative. |  |
| Case: 1 | 42 | M | Upper abdominal; radiating to back | Amylase: 132 U/L (upper: 180 U/L); lipase: 382 (upper: 180 U/L) | Supportive CT scan findings | No respiratory symptoms; COVID-19 test positive 2 days following hospitalization | Cholelithiasis  Alcohol consumption  Hypertriglyceridemia  Hypercalcemia | Likely Nosocomial COVID-19 | [44]  32959016 |
| Case: 2 | 35 | M | Upper abdominal; radiating to back | Amylase: within normal limits; lipase: 1,042 U/L (upper normal limit: 180 U/L) | Supportive CT scan findings | No respiratory symptoms; COVID-19 test positive 1 day following hospitalization | Cholelithiasis  Alcohol consumption  Hypertriglyceridemia  Hypercalcemia  Offending medications | Likely nosocomial COVID-19 |  |
| Case: 1 | 38 | M | No pain reported | No initial amylase or lipase reported | Supportive CT scan findings | 2 weeks prior to respiratory symptoms; patient was hospitalized for 14 days |  | Likely nosocomial COVID-19; alcohol consumption might be the etiology of acute pancreatitis per history | [45]  33005274 |

F, female; M, male; US, ultrasound; EUS, endoscopic ultrasound; CT scan, computed tomography; MRI: magnetic resonance imaging.

**Supplementary References**

1. Hadi, A., et al., *Coronavirus Disease-19 (COVID-19) associated with severe acute pancreatitis: Case report on three family members.* Pancreatology, 2020. **20**(4): p. 665-667.

2. Anand, E.R., et al., *Acute pancreatitis in a COVID-19 patient.* Br J Surg, 2020. **107**(7): p. e182.

3. Aloysius, M.M., et al., *COVID-19 presenting as acute pancreatitis.* Pancreatology, 2020. **20**(5): p. 1026-1027.

4. Meireles, P.A., et al., *Acalculous Acute Pancreatitis in a COVID-19 Patient.* Eur J Case Rep Intern Med, 2020. **7**(6): p. 001710.

5. Suchman, K., et al., *Acute pancreatitis in children hospitalized with COVID-19.* Pancreatology, 2020.

6. Stevens, J.P., et al., *COVID-19-Associated Multisystem Inflammatory Syndrome in Children Presenting as Acute Pancreatitis.* J Pediatr Gastroenterol Nutr, 2020.

7. Samies, N.L., A. Yarbrough, and S. Boppana, *Pancreatitis in Pediatric Patients with COVID-19.* J Pediatric Infect Dis Soc, 2020.

8. Meyers, M.H., et al., *A Case of COVID-19-Induced Acute Pancreatitis.* Pancreas, 2020. **49**(10): p. e108-e109.

9. Kandasamy, S., *An unusual presentation of COVID-19: Acute pancreatitis.* Ann Hepatobiliary Pancreat Surg, 2020. **24**(4): p. 539-541.

10. Acherjya, G.K., et al., *Acute pancreatitis in a COVID-19 patient: An unusual presentation.* Clin Case Rep, 2020. **8**(12): p. 3400-3407.

11. Gadiparthi, C., et al., *Hyperglycemia, Hypertriglyceridemia, and Acute Pancreatitis in COVID-19 Infection: Clinical Implications.* Pancreas, 2020. **49**(7): p. e62-e63.

12. Kumaran, N.K., B.K. Karmakar, and O.M. Taylor, *Coronavirus disease-19 (COVID-19) associated with acute necrotising pancreatitis (ANP).* BMJ Case Rep, 2020. **13**(9).

13. Ahmed, A.O.E., et al., *Acute abdomen -like-presentation associated with SARS-CoV-2 infection.* IDCases, 2020. **21**: p. e00895.

14. Akkus, C., et al., *Development of pancreatic injuries in the course of COVID-19.* Acta Gastroenterol Belg, 2020. **83**(4): p. 585-592.

15. Alloway, B.C., et al., *Suspected case of COVID-19-associated pancreatitis in a child.* Radiol Case Rep, 2020. **15**(8): p. 1309-1312.

16. Alves, A.M., et al., *SARS-CoV-2 leading to acute pancreatitis: an unusual presentation.* Braz J Infect Dis, 2020. **24**(6): p. 561-564.

17. Alwaeli, H., et al., *A Case of Severe Acute Pancreatitis Secondary to COVID-19 Infection in a 30-Year-Old Male Patient.* Cureus, 2020. **12**(11): p. e11718.

18. Bokhari, S. and F. Mahmood, *Case Report: Novel Coronavirus-A Potential Cause of Acute Pancreatitis?* Am J Trop Med Hyg, 2020. **103**(3): p. 1154-1155.

19. Brikman, S., et al., *Acute pancreatitis in a 61-year-old man with COVID-19.* Cmaj, 2020. **192**(30): p. E858-e859.

20. Bruno, G., et al., *Pancreatic injury in the course of coronavirus disease 2019: A not-so-rare occurrence.* J Med Virol, 2020.

21. Cerda-Contreras, C., et al., *Baricitinib as treatment for COVID-19: friend or foe of the pancreas?* Clin Infect Dis, 2020.

22. Cheung, S., A. Delgado Fuentes, and A.D. Fetterman, *Recurrent Acute Pancreatitis in a Patient with COVID-19 Infection.* Am J Case Rep, 2020. **21**: p. e927076.

23. Dietrich, C.G., et al., *Primary presentation of COVID-19 solely with gastrointestinal symptoms: a problem for the containment of the disease.* Eur J Gastroenterol Hepatol, 2020. **32**(11): p. 1475-1478.

24. Fernandes, D.A., A.S. Yumioka, and H.R.M. Filho, *SARS-CoV-2 and acute pancreatitis: a new etiological agent?* Rev Esp Enferm Dig, 2020. **112**(11): p. 890.

25. Gonzalo-Voltas, A., C.U. Fernández-Pérez-Torres, and J.M. Baena-Díez, *Acute pancreatitis in a patient with COVID-19 infection.* Med Clin (Barc), 2020. **155**(4): p. 183-184.

26. Handaya, A.Y., et al., *Covid-19 mimicking symptoms in emergency gastrointestinal surgery cases during pandemic: A case series.* Int J Surg Case Rep, 2020. **77**: p. 22-27.

27. Hassani, A.H., et al., *Unusual gastrointestinal manifestations of COVID-19: two case reports.* Gastroenterol Hepatol Bed Bench, 2020. **13**(4): p. 410-414.

28. Karimzadeh, S., et al., *COVID-19 presenting as acute pancreatitis: Lessons from a patient in Iran.* Pancreatology, 2020. **20**(5): p. 1024-1025.

29. Kataria, S., et al., *COVID-19 Induced Acute Pancreatitis: A Case Report and Literature Review.* Cureus, 2020. **12**(7): p. e9169.

30. Kurihara, Y., et al., *Pancreatitis in a Patient with Severe Coronavirus Disease Pneumonia Treated with Veno-venous Extracorporeal Membrane Oxygenation.* Intern Med, 2020. **59**(22): p. 2903-2906.

31. Lakshmanan, S. and A. Malik, *Acute Pancreatitis in Mild COVID-19 Infection.* Cureus, 2020. **12**(8): p. e9886.

32. Liaquat, H., et al., *High-Dose Prednisone for Treatment of Autoimmune Pancreatitis in a Patient with Coronavirus Disease 2019 (COVID-19) due to Infection with Severe Acute Respiratory Syndrome Coronavirus 2 (SARS-CoV-2).* Am J Case Rep, 2020. **21**: p. e926475.

33. Marchi, G., et al., *Cytomegalovirus-Induced Gastrointestinal Bleeding and Pancreatitis Complicating Severe Covid-19 Pneumonia: A Paradigmatic Case.* Mediterr J Hematol Infect Dis, 2020. **12**(1): p. e2020060.

34. Mazrouei, S.S.A., G.A. Saeed, and A.A. Al Helali, *COVID-19-associated acute pancreatitis: a rare cause of acute abdomen.* Radiol Case Rep, 2020. **15**(9): p. 1601-1603.

35. Miao, Y., O. Lidove, and W. Mauhin, *First case of acute pancreatitis related to SARS-CoV-2 infection.* Br J Surg, 2020. **107**(8): p. e270.

36. Narang, K., et al., *Acute Pancreatitis in a Pregnant Patient With Coronavirus Disease 2019 (COVID-19).* Obstet Gynecol, 2020. **Publish Ahead of Print**.

37. Niño-Taravilla, C., et al., *Pediatric Inflammatory Multisystem Syndrome Temporally Associated with SARS-CoV-2 Treated with Tocilizumab.* Pediatr Rep, 2020. **12**(3): p. 142-148.

38. Patnaik, R.N.K., A. Gogia, and A. Kakar, *Acute pancreatic injury induced by COVID-19.* IDCases, 2020. **22**: p. e00959.

39. Pinte, L. and C. Baicus, *Pancreatic involvement in SARS-CoV-2: case report and living review.* J Gastrointestin Liver Dis, 2020. **29**(2): p. 275-276.

40. Purayil, N., et al., *COVID-19 Presenting as Acute Abdominal Pain: A Case Report.* Cureus, 2020. **12**(8): p. e9659.

41. Rabice, S.R., et al., *COVID-19 infection presenting as pancreatitis in a pregnant woman: A case report.* Case Rep Womens Health, 2020. **27**: p. e00228.

42. Shinohara, T., et al., *Acute Pancreatitis During COVID-19 Pneumonia.* Pancreas, 2020. **49**(10): p. e106-e108.

43. Szatmary, P., et al., *Emerging Phenotype of Severe Acute Respiratory Syndrome-Coronavirus 2-associated Pancreatitis.* Gastroenterology, 2020. **159**(4): p. 1551-1554.

44. Wang, K., et al., *Acute Pancreatitis as the Initial Manifestation in 2 Cases of COVID-19 in Wuhan, China.* Open Forum Infect Dis, 2020. **7**(9): p. ofaa324.

45. Zielecki, P., et al., *Effective treatment of severe acute pancreatitis and COVID-19 pneumonia with tocilizumab.* Prz Gastroenterol, 2020. **15**(3): p. 267-272.
